# Long-Term Variations in Global Solar Radiation and Its Interaction with Atmospheric Substances at Qomolangma

**DOI:** 10.3390/ijerph19158906

**Published:** 2022-07-22

**Authors:** Jianhui Bai, Xuemei Zong, Yaoming Ma, Binbin Wang, Chuanfeng Zhao, Yikung Yang, Jie Guang, Zhiyuan Cong, Kaili Li, Tao Song

**Affiliations:** 1 LAGEO, Institute of Atmospheric Physics, Chinese Academy of Sciences, Beijing 100029, China; zongxm@mail.iap.ac.cn; 2 State Key Laboratory of Tibetan Plateau Earth System, Resources and Environment (TPESRE), Institute of Tibetan Plateau Research, Chinese Academy of Sciences, Beijing 100101, China; ymma@itpcas.ac.cn (Y.M.); wangbinbin@itpcas.ac.cn (B.W.); zhiyuancong@itpcas.ac.cn (Z.C.); 3 College of Earth and Planetary Sciences, University of Chinese Academy of Sciences, Beijing 100049, China; 4 College of Atmospheric Science, Lanzhou University, Lanzhou 730000, China; 5 National Observation and Research Station for Qomolongma Special Atmospheric Processes and Environmental Changes, Dingri, Shigatse 858200, China; 6 Kathmandu Center of Research and Education, Chinese Academy of Sciences, Beijing 100101, China; 7 China-Pakistan Joint Research Center on Earth Sciences, Chinese Academy of Sciences, Islamabad 45320, Pakistan; 8 State Key Laboratory of Earth Surface Processes and Resource Ecology, College of Global Change and Earth System Science, Beijing Normal University, Beijing 100875, China; czhao@bnu.edu.cn (C.Z.); ykyang@mail.bnu.edu.cn (Y.Y.); 9 State Environment Protection Key Laboratory of Satellite Remote Sensing, Aerospace Information Research Institute, Chinese Academy of Sciences, Beijing 100101, China; guangjie@aircas.ac.cn; 10 Nanjing Zhongkehuaxing Emergency Science and Technology Research Institute, Nanjing 211899, China; 20171201073@nuist.edu.cn (K.L.); tao_song@aliyun.com (T.S.)

**Keywords:** absorbing and scattering, energy, air temperature, wind speed, climate and climate change

## Abstract

An empirical model to estimate global solar radiation was developed at Qomolangma Station using observed solar radiation and meteorological parameters. The predicted hourly global solar radiation agrees well with observations at the ground in 2008–2011. This model was used to calculate global solar radiation at the ground and its loss in the atmosphere due to absorbing and scattering substances in 2007–2020. A sensitivity analysis shows that the responses of global solar radiation to changes in water vapor and scattering factors (expressed as water-vapor pressure and the attenuation factor, AF, respectively) are nonlinear, and global solar radiation is more sensitive to changes in scattering than to changes in absorption. Further applying this empirical model, the albedos at the top of the atmosphere (TOA) and the surface in 2007–2020 were computed and are in line with satellite-based retrievals. During 2007–2020, the mean estimated annual global solar radiation increased by 0.22% per year, which was associated with a decrease in AF of 1.46% and an increase in water-vapor pressure of 0.37% per year. The annual mean air temperature increased by about 0.16 °C over the 14 years. Annual mean losses of solar radiation caused by absorbing and scattering substances and total loss were 2.55, 0.64, and 3.19 MJ m^−2^, respectively. The annual average absorbing loss was much larger than the scattering loss; their contributions to the total loss were 77.23% and 22.77%, indicating that absorbing substances play significant roles. The annual absorbing loss increased by 0.42% per year, and scattering and total losses decreased by 2.00% and 0.14% per year, respectively. The estimated and satellite-derived annual albedos increased at the TOA and decreased at the surface. This study shows that solar radiation and its interactions with atmospheric absorbing and scattering substances have played key but different roles in regional climate and climate change at the three poles.

## 1. Introduction

The global climate and atmospheric substances have been changing faster in recent decades. The air temperature is increasing in most areas on the Earth, along with increases in gases, liquids, and particles (GLPs) emitted by anthropogenic and biogenic sources and produced by chemical and photochemical reactions (CPRs), during which solar ultraviolet radiation (UV) and visible radiation (VIS) are absorbed and used by GLPs. Over each region on the Earth, solar radiation is an important energy source driving the atmosphere and interactions in the atmosphere, biosphere, and land/sea, thus controlling the regional energy balance at the top of the atmosphere (TOA) and the surface of land and/or sea and ultimately controlling horizontal and vertical air movement in the atmosphere and the regional climate. These processes (in the emissions and formation of GLPs, radiative transfer, CPRs, etc.) influence the global climate system through energy and GLP exchanges in different regions by different mechanisms, including transport processes in the troposphere and stratosphere [1,2,3,4,5,6,7,8,9,10], exchange between the troposphere and stratosphere, and regional and global circulation. Therefore, more detailed studies should be conducted on the above processes.

The Himalayas is a channel for the exchange of energy and materials between the surface and tropospheric atmosphere [11], and strong diurnal variations in valley winds exist in the north valley of the Qomolangma Mountains [12]. The Tibetan Plateau (TP, Qinghai–Xizang Plateau, China), covering about one-quarter of Chinese territory, is the largest plateau in the world and has profound thermal and dynamic influences on atmospheric circulation over East Asia and even the whole Northern Hemisphere [13]. The Tibetan Plateau is a source of heat in the atmosphere in summer [13] and plays an important role in weather and climate systems. The Tibetan Plateau, as the world’s third pole with high altitude, is a vulnerable and sensitive region with a strong response to global climate change [14]. Several studies have reported that the air temperature over the TP is also increasing [15,16,17]; e.g., the air temperature in February increased by 0.34 °C/decade from 1951–2013 over the TP ([18] and references therein) and by 0.33 °C/decade from 1961–2018 over the Qomolangma region [19]. Carbon dioxide, black carbon (BC), and greenhouse gases may be sufficient to account for a warming trend of 0.25 °C per decade in the Himalayas [14].

Global warming, including in the Antarctic and Arctic, during the 20th century is reported by the Intergovernmental Panel on Climate Change (IPCC) and other studies [20,21,22,23,24,25,26,27], and anthropogenic increases in greenhouse gases (GHGs) are considered a common cause, besides astronomical factors [28]. There is still a great need to investigate the reasons and mechanisms associated with regional and global climate and climate change. Solar radiation, as an important energy source, controls the changes in and movements of atmospheric substances, as well as climate change. Therefore, solar radiation transfer along with its transformation processes (absorption, scattering, reflection, etc.) in the atmosphere and at the TOA and the surface should be fully studied using surface- and satellite-based observations and model estimations.

A large number of models, including radiative transfer and empirical models, widely applied for calculating global, direct, and diffuse solar radiation at the surface, are presented in [9,29,30,31,32,33,34,35,36,37,38]. A previous empirical model of global solar radiation used at Sodankylä in the Arctic was further developed at the Qomolangma site, TP. The main objectives of this paper are to study (1) the characteristics of global solar radiation (G) at the surface, (2) the losses of G in the atmosphere caused by absorbing and scattering substances and their relative contributions to the total loss, (3) albedos at the TOA and at the surface, (4) the relationships between absorbing and scattering radiation and their driving factors, and (5) long-term variations in and interactions among the above parameters.

The results at four typical sites, Qomolangma, Sodankylä (67.367 N, 26.630 E, 184 m) in the Arctic, Qianyanzhou (26°44′48′ N, 115°04′13′ E, 110.8 m, a subtropical *Pinus* forest, China) at mid-latitude in the Northern Hemisphere, and Dome C (75°06′ S, 123°21′ E, 3233 m) in the Antarctic, were compared to fully understand the basic processes and mechanisms in the interactions between solar radiation, atmospheric substances, and the climate.

## 2. Data and Methodology

### 2.1. Measurements and Data Usage

The Qomolangma Atmospheric and Environmental Observation and Research Station, Chinese Academy of Sciences (QOMS, 28.21° N, 86.56° E, 4276 m), is located in Dingri County, TP, in the north region of Mt. Qomolangma (Everest). The surrounding surface is flat and mainly covered by sand and gravel, as well as sparse and short vegetation [11,39]. Solar radiation and meteorological parameters [11] were measured routinely at QOMS Station. Downward and upward global solar radiations were measured by pyranometers (model CM21, Kipp & Zonen Inc., Delft, The Netherlands). Air temperature (T), relative humidity (RH), and wind speed (v) were measured by HMP45C-GM (Vaisala Inc., Vantaa, Finland) and 034B (Met One Inc., Grants Pass, OR, USA), respectively. An hourly dataset from January 2007 to December 2020 was used in this study. More detailed information about the instruments and station is reported in [11,39].

To ensure the high quality of observational data, measured hourly global solar radiation larger than 50 W m^−2^ was used in this study, including hourly, daily, and monthly averages. Extremely high hourly radiation (e.g., 2 times higher than the maximum at the corresponding time under clear skies) and extinction/attenuation factors (AF, Section 2.2) were removed, and there were much stricter criteria than those used in other studies [40]. When G is <50 W m^−2^_,_ the solar altitude angle is very low, causing larger measurement errors in G and AF. Firstly, an empirical model of G under all-sky conditions was developed using hourly global solar radiation and meteorological variables during 2008–2011, which was the same time period as that used in the empirical model development for the two poles at Sodankylä and Dome C. Secondly, it was applied to calculate G and its loss in the atmosphere and reflections/albedos at the TOA and the surface during 2007–2020. Thirdly, multiple interactions between solar radiation energy (absorption, scattering, etc.), atmospheric substances, and meteorological variables were investigated.

### 2.2. Empirical Model Development and Validation

Solar radiation is transferred in the atmosphere and mainly takes part in two processes, absorption and scattering, which are described in an empirical model [9,41]: (1) The photochemical term, the absorption of G by atmospheric substances, is calculated using the extinction term e^−kWm^ × cos(Z), where k is the mean absorption coefficient of water vapor, W is the water-vapor content in the atmospheric column (cm), m is the optical air mass, and Z is the solar zenith angle. W = 0.02 × E × 60, where E is the water-vapor pressure at the ground (hPa). e^−kWm^ = 1 − 0.17 × (m × W)^0.30^/I_0_, where I_0_ = 1.94 cal^−1^ min^−2^ (1367 W m^−2^). This term expresses GLP absorption and indirect use in CPRs through OH radicals, H_2_O, and volatile organic compounds (VOCs) by different mechanisms in the short-wavelength region (i.e., UV, VIS, and near-infrared, NIR); more detailed explanations about this term and the associated mechanisms are reported in Section 2.2 and Section 4.1 in [9] (and related papers [20,22] therein). (2) The scattering term, the scattering of G caused by atmospheric substances, is calculated using e^−AF^. An empirical model was determined to calculate G under all-sky conditions [9,38]:

G = A_1_e^−kWm^ × cos(Z) + A_2_e^−AF^ + A_0_ (1)

AF = (1 − (G/cos(Z) − G_Dmax_))/(G_Dmax_/G_Mmax_)/(G_Mmax_/G_Ymax_)/(G_Ymax_/G_4Ymax_) (2)

where G is hourly global solar radiation at the surface (MJ m^−2^); G_Dmax_, G_Mmax_, and G_Ymax_ are the maxima of G for each day, month, and year, respectively; and G_4Ymax_ is the maximum G in four years (2008–2011). The attenuation factor represents the total extinction of global solar radiation, i.e., including absorption and scattering. A_1_ and A_2_ (MJ m^−2^) are the individual absorbing and scattering solar radiation associated with the absorbing and scattering GLPs at the TOA, and A_0_ (MJ m^−2^), a negative value, is the reflection of G at the TOA. The sum of A_1_, A_2_, and A_0_ represents G at the TOA and should be equal to the widely used solar constant (I_0_ = 4.92 MJ m^−2^). A_1_, A_2_, and A_0_ were objectively determined by analyzing the observational data based on Equations (1) and (2).

To obtain an empirical model that reveals a reliable relationship between physical and chemical processes under all-sky conditions with more high-quality observational data (Z < 55°, small observational errors, and sample number n = 7020), 7020 hourly data points from January 2008 to December 2011 were used for model development, and the usage rate of the observational data was 45.19%. Hourly averages of solar radiation and meteorological parameters (i.e., T, RH, and E) were used to calculate daily and monthly averages [42].

**Figure 1:**
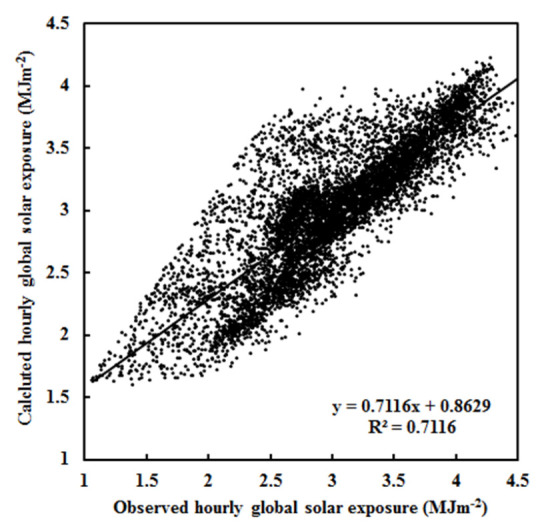
Scatter plot of calculated versus observed hourly global solar exposure under all-sky conditions at Qomolangma (n = 7020).

All coefficients in Equation (1) were determined by using a multi-parameter fit to the measured hourly G. The results are shown in Table 1, including the optimized coefficients (A_i_), the coefficient of determination (R^2^), the mean absolute value of relative error (δ) between calculated and measured G, the mean absolute deviation (MAD, in exposure units, MJ m^−2^, and in the percentage of mean measured value, %), and the root mean square error (RMSE, in exposure units and in the percentage of mean measured value). Figure 1 displays a scatter plot of calculated versus observed hourly G. The empirical model is in reasonable agreement with measurements under all-sky conditions.

When analyzing observed hourly data (n = 7020), there was a strong correlation between G and absorbing and scattering terms (R = 0.844, at a confidence level α = 0.001). The correlation between G and the absorption term (R = 0.777) was much stronger than the correlation between G and the scattering term (R = 0.037), while a weak correlation existed between the absorption and the scattering terms (R = 0.348). Thus, the absorbing and scattering processes are well distinguished by the empirical model, and water-vapor pressure and the attenuation factor can represent the absorbing and scattering substances, respectively. A similar result was also found in a previous study [9]. Thus, the attenuation factor is also called the scattering factor. The RMSE (0.31 MJ m^−2^, Table 1) was a little larger than or close to the mean RMSE (0.22) obtained using 7 independent a priori models with the best performance out of 105 empirical models [34].

**Figure 2:**
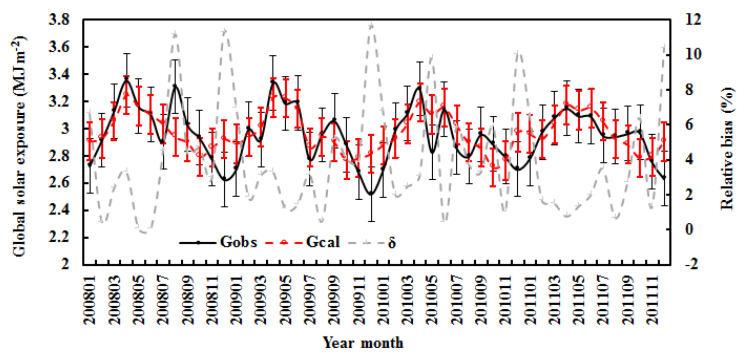
The observed and calculated monthly averages of hourly global solar exposure (G_obs_ and G_cal_) and relative biases (δ) in all-sky conditions at the Qomolangma site. The error bars show the standard deviations of the observed and calculated values (1σ, red for the calculated values).

**Figure 3:**
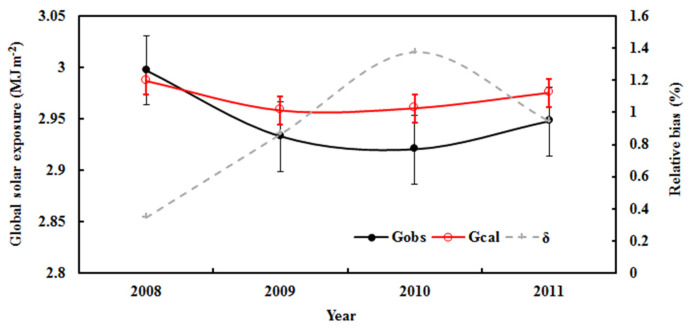
The calculated and observed annual averages of hourly global solar exposure (G_obs_ and G_cal_) and relative biases (δ) in all-sky conditions at the Qomolangma site. The error bars show the standard deviations of the observed and calculated values (1σ, red for the calculated values).

The estimated monthly average G was also in agreement with the measurements, with a relative bias of 3.96% for the average and 11.64% for the maximum values (Figure 2). The RMSE values were 0.15 MJ m^−2^ and 4.92%. The above corresponding values for annual mean G (Figure 3) were 0.88% and 1.37% (relative bias) and 0.03 MJ m^−2^ and 1.09% (RMSE). Both the estimated and measured annual mean G decreased by 0.11% and 0.54% per year, respectively, during 2008–2011. The above results show that the empirical model performs reliable simulations.

To evaluate the empirical model in performance testing, firstly, the observed hourly global solar radiation for Z < 75° and G ≥ 20 Wm^−2^ from January to December from 2008–2011 were used. The mean absolute relative bias was 60.14%, and the NMSE was 0.083. The RMSE values were 0.58 MJ m^−2^ and 28.88% (n = 15,533). Figure 2 displays the monthly averages of estimated and observed G. 

It has been reported that the uncertainties of many solar radiation models are as high as 20% [43]. In general, the calculated global solar radiation overestimated the observed value, as the empirical model was established for optimum atmospheric conditions (i.e., high G_obs_). The estimated monthly average of G was also in line with the measured value, with a relative bias of 7.13% (average) and 24.95% (maximum). The RMSE values were 0.18 MJ m^−2^ and 9.10%. The standard deviations of estimated and measured G were 0.17 and 0.19 MJ m^−2^ (Figure 3). The annual averages of estimated and measured G showed similar variations, with a relative bias of 2.72% (average) and 3.81% (maximum). The RMSE values were 0.07 MJ m^−2^ and 3.50%. The standard deviations of estimated and observed G were 0.05 and 0.03 MJ m^−2^.

Secondly, to further evaluate the empirical model, the observed hourly G for Z < 75° and G ≥ 50 Wm^−2^ from January to December in 2008–2011 were used (n = 14,886). The calculated results, together with statistical metrics, are shown in Table 2. 

Generally, this evaluation produced better estimations of hourly, monthly, and annual averages of G, with smaller values of δavg, NMSE, σ, MAD, and RMSE. For example, δavg = 40.07%, and RMSE = 0.56 MJ m^−2^ and 27.10%, respectively.

Thirdly, observed hourly data (G > 100 W m^−2^ and Z < 75°) from 1 January 2007 to 31 December 2020, except for 1 January 2008 to 31 December 2011 (used in model development) under all-sky conditions were used for model evaluation (n = 32,976). The results showed that δavg = 22.42%, NMSE = 0.053 MJ m^−2^, MAD = 0.368 MJ m^−2^ and 17.11%, RMSE = 0.495 MJ m^−2^ and 23.19%, σ_cal_ = 1.03, and σ_obs_ = 0.98. These results indicate that the empirical model performs better simulations with larger G (i.e., 100 versus 50 and 20 Wm^−2^) and higher-accuracy observations (i.e., lower σ_cal_ and σ_obs_).

Comparing RMSE values of the empirical model in this study and others, with direct normal irradiance as a relative reference, our above three RMSE values were larger than that for an arid climate zone (ranging from 15.1% to 31.0%) and lower than those of 8 of the 9 best models (among 140) for temperate (28.2–40.3%) and tropical climate (32.0–51.8%) zones [44].

All of the above results indicate that the empirical model provides reasonable estimates of hourly, monthly, and annual G under all-sky conditions.

## 3. Results

### 3.1. Global Solar Radiation during 2007–2020

To study the basic characteristics of G and meteorological parameters at Qomolangma, observed hourly data from 1 January 2007 to 31 December 2020 were analyzed. To obtain the best representatives of the idealized atmospheric states that can be used to further investigate interactions and mechanisms among solar radiation, atmospheric GLPs, meteorological variables, and climate and climate change, observed hourly G > 100 W m^−2^ and Z < 75° were used in the analysis.

During the daytime in 2007–2021, the averages of observed hourly G (n = 46,683) were 2.16 MJ m^−2^ (corresponding to 599.04 W m^−2^). The mean AF was 2.97, and averages of air temperature, relative humidity, and water-vapor pressure were 8.03 °C (ranging from −24.77 to 34.20 °C), 32.69% (0–100%), and 3.85 hPa (0.06–45.77 hPa), respectively. Similarly, the average air pressure and wind speed were 604.94 hPa (588.17–615.10 hPa) and 3.50 ms^−1^ (0–16.08 ms^−1^), respectively.

Hourly global radiation was calculated for Qomolangma for 1 January 2007–31 December 2020 using the empirical model and input variables, measured hourly water-vapor pressure at the ground and AF. The calculated and observed hourly global solar radiation varied with similar patterns. The calculated values overestimated the observed value by 21.98% on average, the NMSE was 0.05, and the RMSE values were 0.48 MJ m^−2^ and 22.13% in 2007–2020. These values were larger than the corresponding ones in the model development (n = 7020). This is reasonable because the empirical model represents the global solar radiation and its relationships with absorbing and scattering GLPs under optimal atmospheric conditions (clean atmosphere, low AF = 1.66 during 2008–2011 in model development). In addition, the relative error of 21.98% is close to the uncertainty of 20% of the popular solar radiation models [43].

**Figure 4:**
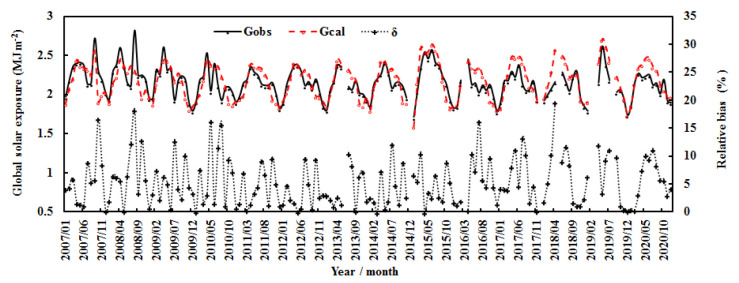
Monthly global solar exposure (calculated and observed) (G) and relative biases (δ) at the Qomolangma site during 2007–2020.

**Figure 5:**
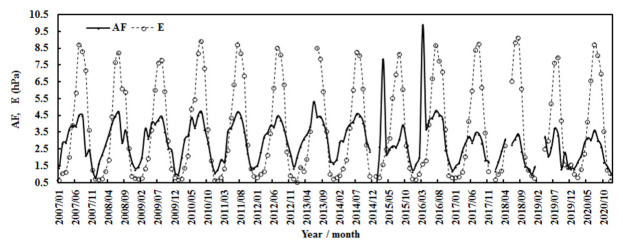
Monthly water-vapor pressure (E) and attenuation (AF) at the Qomolangma site during 2007–2020.

Figure 4 and Figure 5 show the calculated and observed monthly G and its influencing factors, water-vapor pressure (E) and the attenuation factor (AF). Generally, global solar radiation displayed significant seasonal variations and peaked in spring and summer. E and AF also exhibited strong seasonal variations and peaked in summer. The estimated monthly G overestimated the measured value by 5.46%, the NMSE was 0.005, and the RMSE values were 0.15 MJ m^−2^ and 7.05%.

**Figure 6:**
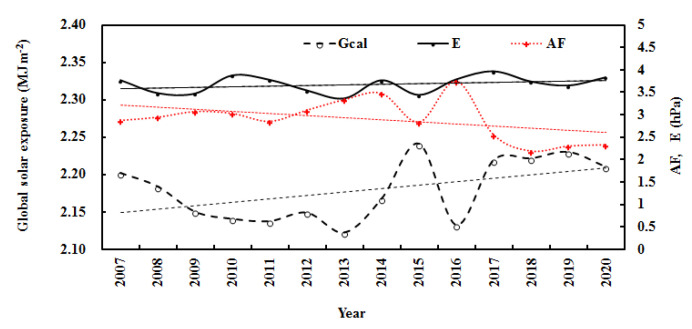
Annual calculated global solar exposures (G_cal_), water-vapor pressure (E), and attenuation factor (AF) at the Qomolangma site during 2007–2020. The lines are linear fits to the data for E (upper line), AF (middle line), and G_cal_ (lower line).

During the 14 years, the monthly mean observed and computed G generally remained stable, E increased by 0.008% per month, and AF decreased by 0.001% per month. The air temperature increased by 0.002% per month (corresponding to 0.47 °C), and relative humidity remained stable. For the annual average, the computed G increased by 0.22% per year, which was attributed to the total contribution from the decrease in AF of 1.46% per year and the increase in E of 0.37% per year (Figure 6). Generally, the annual air temperature increased by 0.16 °C during the 14 years, corresponding to climate warming in this region [15,16,17,18,19], and the annual relative humidity increased by 0.63% per year. The above results indicate that the atmosphere over the Qomolangma region became cleaner and warmer in 2007–2020. This warming phenomenon is in line with the climate warming at Sodankylä in the Arctic during 2000–2018 and at Dome C in the Antarctic during 2006–2016 [38,45], indicating that climate warming has appeared at the three representative sites in the three poles.

Monthly averages of air temperature, relative humidity, and water-vapor pressure showed synchronous variations with clear correlations between T and RH and between T and E (R = 0.820 and 0.896, respectively), and there was a stronger correlation between T and E than between T and RH.

During the 14 years, the annual mean calculated and observed global solar radiation was 2.18 and 2.15 MJ m^−2^, corresponding to 605.21 and 596.26 W m^−2^, respectively, showing that 44.27% and 43.62% of the total solar radiation (G/I_0_) arrived at the ground. The mean estimated annual G was evidently attenuated by atmospheric GLPs and inversely varied with the attenuation factor (AF) (Figure 6). The correlation between G_cal_ and AF was 0.756. To better understand the mean atmospheric conditions during 2007–2020 at Qomolangma, the annual averages of the other variables were calculated and were 2.91 for AF, 7.71 °C for T, 31.95% for RH, 3.67 hPa for E, 3.52 ms^−1^ for wind speed, and 665.79 hPa for air pressure.

Table 3 displays the detailed statistical metrics for estimated and measured G during 2007–2020. In general, the empirical model performed better estimations under the conditions of larger observed G and better-quality data.

### 3.2. The Losses of Global Solar Radiation in the Atmosphere during 2007–2020

The hourly losses of global solar radiation caused by absorbing and scattering substances (G_LA_ and G_LS_) were computed using A_1_(1 − e^−kWm^ × cos(Z)) and A_2_(1 − e^−AF^), respectively, and the total loss G_L_ was G_LA_ + G_LS_. 

**Figure 7:**
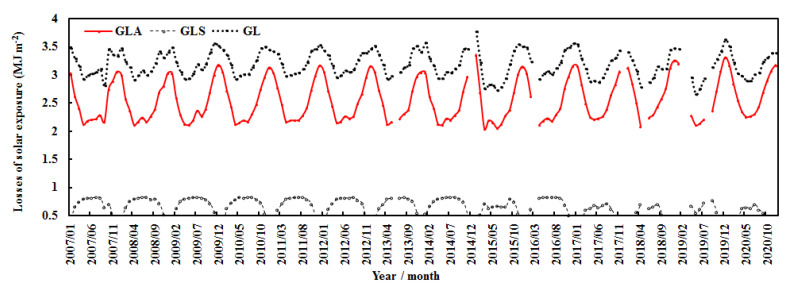
Monthly losses of global solar exposure caused by absorbing and scattering substances (G_LA_ and G_LS_) and total loss (G_L_ = G_LA_ + G_LS_) at the Qomolangma site.

For the monthly average, G_LA_ due to absorbing GLPs dominated the total loss and showed strong seasonal variation with the highest values in winter and lowest values in spring and summer. G_LS_ due to scattering GLPs also displayed clear seasonal variation with peaks in summer and low values in winter. From January 2007–December 2020, (1) the monthly G_LA_ increased by 0.003%, associated with an increase in E of 0.008%; (2) the monthly G_LS_ decreased by 0.002%, associated with a decrease in AF of 0.001%; and (3) the monthly G_L_ remained stable (Figure 7).

**Figure 8:**
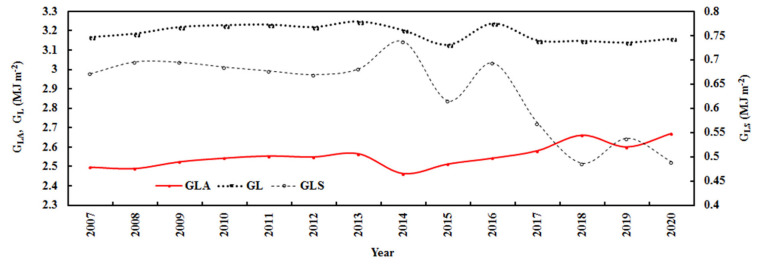
Annual losses of global solar exposure caused by absorbing and scattering substances (G_LA_ and G_LS_) and total loss (G_L_ = G_LA_ + G_LS_) at the Qomolangma site.

**Figure 9:**
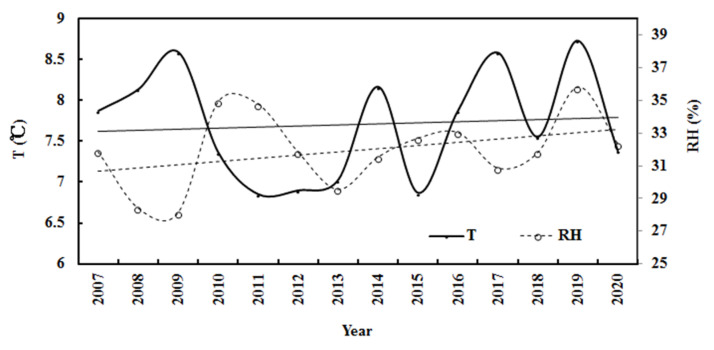
Annual air temperature (T) and relative humidity (RH) at the Qomolangma site during 2007–2020. The lines are linear fits to the data for T (upper line) and RH (lower line).

The annual absorbing, scattering, and total losses showed evident interannual changes (Figure 8), which were associated with changes in E, AF, T, and RH (Figure 6 and Figure 9). G_LA_ increased by 0.42% per year, associated with an increase in E of 0.37%; G_LS_ decreased by 2.00% per year, associated with a decrease in AF of 1.46%; and annual G_L_ decreased by 0.14% per year.

**Figure 10:**
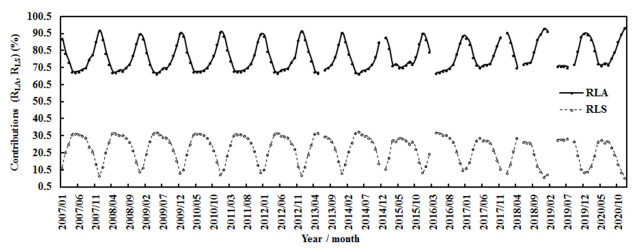
Contributions (R_LA_ and R_LS_) of monthly absorbing and scattering losses to the monthly total loss at the Qomolangma site.

During January 2007–December 2020, the contributions of absorbing and scattering losses (R_LA_ = A_1_(1 − e^−kWm^ × cos(Z))/(A_1_(1 − e^−kWm^ × cos(Z) + A_2_(1 − e^−AF^)), R_LS_ = A_2_(1 − e^−AF^)/(A_1_(1 − e^−kWm^ × cos(Z) + A_2_(1 − e^−AF^)) to the total loss were 77.19% and 22.81% for the monthly average, respectively (Figure 10), corresponding to a monthly mean E of 3.67 hPa, AF of 2.92, and T of 7.69 °C. Generally, R_LA_ was higher in winter and lower in spring and summer (e.g., April–August), revealing that the absorbing constituents/processes dominated the attenuation of global solar radiation, while R_LS_ varied inversely compared to R_LA_ (most peaks occurred in spring and summer, and the lowest values were in winter). The above corresponding absorbing and scattering contributions (R_LA_ and R_LS_) were 77.23% and 22.77% for the annual average, respectively.

During 2007–2020, the annual mean monthly losses of G_LA_, G_LS_, and G_L_ were 2.55, 0.64, and 3.19 MJ m^−2^, respectively, and reflection at TOA (i.e., A_0_) was 1.01 MJ m^−2^, corresponding to 709.41, 177.18, 886.59, and 280.74 W m^−2^, respectively.

### 3.3. Analysis of Global Solar Radiation and Its Loss and Meteorological Variables in the Periods of Model Development and 2007–2020

To thoroughly investigate the variation in and mechanism of solar radiation, climate, and their interactions, as well as monthly mean global solar radiation and absorbing and scattering substances/factors (E and AF), meteorological variables (air temperature T, air pressure p, and wind speed v) were calculated, along with their cross-correlations for three time periods: January 2008–December 2011 (model development, situation A, n = 7020), January 2008–December 2011 (the whole measured dataset, situation B, n = 14,886), and January 2007 to December 2020 (situation C, n = 46,683). 

The change rates of the monthly mean solar radiation and meteorological parameters are presented in Table 4. Both the calculated and observed monthly G decreased, which was attributed to the strict criteria for data usage in model development. Better performance of the empirical model of G was also found at the other two polar sites and a site in a mid-latitude region, with close estimations of G and similar change rates of calculated and observed G [9,38,45]. Comparing the calculations using the same dataset used in model development and other datasets in all-sky conditions at the above four sites, the estimates of G at Qomolangma were a little worse. For example, more scattered points appeared in the scatter plot (e.g., Figure 1), which was mainly caused by larger observational errors. Thus, the estimated G_cal_ was selected for the subsequent analysis.

In situation A, the monthly G_cal_ decreased, the monthly losses G_LA_ and G_L_ increased, and G_LS_ decreased. T decreased, and E and AF increased. The air temperature reduction of 1.45 °C was the result of the decrease in G_cal_ at the surface, which leads to a decrease in the long-wave radiative heating of the atmosphere, and the decrease in scattering loss G_LS_, i.e., the loss of scattering energy, associated with the loss in atmospheric internal energy.

In situation B, the monthly G_cal_ increased, G_LA_ increased, and G_LS_ and G_L_ decreased. T decreased (0.70 °C), and E and AF increased. The larger temperature drop in situation A was caused by decreases in both G_cal_ and G_LS,_ while the temperature drop in situation B was caused by only the decrease in G_LS_.

In situation C, the monthly G_cal_ increased a little (or remained stable), G_LA_ increased, and G_LS_ decreased. T and E increased, but AF decreased. This reveals that the atmosphere was warming at Qomolangma during 2007–2020, which was caused by increases in both the absorbing energy in the atmosphere and G_cal_ at the surface, some of which can be converted to long-wave radiation. The increases in atmospheric absorbing GLPs (expressed as E), which are mainly gases, as particles represented by decreased AF, are the critical contributing factors under all-sky conditions. It should be noted that the absorbing GLPs in the UV, VIS, and NIR regions include all GHGs and non-GHGs [9]. 

Therefore, air temperature changes mainly depend on atmospheric substances and their changes, as well as the losses of global solar radiation and G received at the ground. GLPs with strong absorption in the NIR region also play important roles.

To comprehensively understand the interactions and mechanisms between solar radiation and atmospheric variables, correlations among the above parameters in January 2008–December 2011 (model development, n = 7020) were computed (Table 5). Strong and positive correlations were found between T and observed and calculated G, T and G_LS_, T and E, T and AF, G_LS_ and AF, and P and AF (α = 0.001), and negative correlations existed between T and G_LA_, G_LA_ and E, and v and AF (α = 0.001). These correlation results suggest at least the following five points. (1) Air temperature is positively influenced by G received at the ground that is attenuated by absorbing and scattering GLPs in the atmosphere. (2) Absorbing and scattering GLPs (described by E and AF) play significant roles in absorbing and scattering processes and losses, respectively. Higher consistency appeared in the variations between G_LS_ and AF than between G_LA_ and AF. (3) The GLPs in the whole atmospheric column make positive contributions to air pressure, and absorbing GLPs are more important than scattering GLPs. (4) Scattering energy lost in the atmosphere has a slightly greater (positive) contribution to the air temperature (representing atmospheric internal energy) increase and its change than absorbing energy. (5) The absorbing and scattering GLPs make positive contributions to the air temperature increase through different mechanisms in short- and long-wave solar radiation. It should be noted that water vapor is one of the important GHGs (a strong correlation R = 0.569, α = 0.001, existed between T and E).

The increase in monthly air temperature (0.47 °C) in 2007–2020 was mainly caused by increases in estimated G that converted to long-wave radiation heating of the atmosphere, partial G_LA_, and water vapor. In contrast, air temperature decreased in situation A, revealing a mechanism for the energy losses of heating the atmosphere: (1) G_cal_ (representing the long-wave radiation emitted from the ground) and (2) G_LS_ (representing collision energy from photons) under clearer atmospheric conditions (e.g., low AF) than situations B and C. Therefore, air temperature and its change depend on different radiation energy, total atmospheric GLPs, and their complicated and multiple interactions.

The contributions of absorbing and scattering losses to the total loss were about 72.54% and 27.46% for hourly values in model development (n = 7020), respectively, which are similar to the corresponding values during 2007–2020. However, the scattering processes made a slightly greater contribution under cleaner atmosphere conditions (lower AF, n = 7020), revealing that small-sized aerosols produced through CPRs under high G and T make larger contributions.

The hourly averages of solar radiation and meteorological parameters during January 2008–December 2011 (model development, situation A, n = 7020), January 2008–December 2011 (whole measured dataset, situation B, n = 14,886), and January 2007 to December 2020 (situation C, n = 46,683) are given in Table 6. Comparing situation A with situations B and C (i.e., clean atmosphere to high GLP loads), small absorption, scattering, and total energy losses in the atmosphere under cleaner atmospheric conditions (A) were associated with lower absorbing and scattering GLPs (represented by E and AF, respectively). The higher the GLP loads, the larger the absorbing energy lost; i.e., the larger R_LA_ was contributed to by absorbing processes in the atmosphere (e.g., 77% versus 73%), revealing that absorbing GLPs play dominant roles in radiative transfer at Qomolangma. Air temperature was the highest in situation A, followed by situations C and B, closely corresponding to G at the ground, revealing that long-wave radiation converted from the ground is a significant energy source heating the atmosphere and causing regional climate warming. More studies on the mechanism of air temperature change due to long-wave radiation are suggested.

### 3.4. Sensitivity Analysis

**Figure 11:**
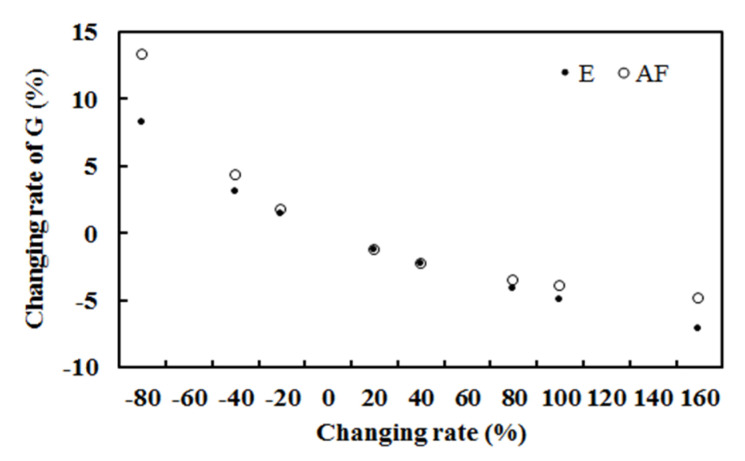
Change rates of G (%) due to changes in E or AF (%), with AF or E retaining their original values.

The response of the computed hourly global solar radiation to changes in absorbing or scattering GLPs (represented by E and AF, respectively) was investigated using the empirical model (Equation (1), n = 7020), with other factors kept at their original levels. The results are shown in Figure 11 and Table 7. The responses of G_cal_ to changes in both E and AF were nonlinear and negative.

The estimated G at the ground increased/decreased with the decrease/increase in water vapor, meaning that the increase in absorbing GLPs results in a large loss of global solar radiation in the atmosphere and at the ground. In addition, G_cal_ increased/decreased with the decrease/increase in scattering GLPs, indicating that the increase in scattering GLPs leads to a large attenuation of global solar radiation. G_cal_ was more sensitive to changes in the scattering factor (AF) than the absorbing factor (E) when E and AF decreased, revealing that much more scattering energy was lost in the atmosphere than absorption energy (at a very low E of 3.75 hPa, n = 7020). G_cal_ was more sensitive to changes in E than AF when E and AF increased, revealing that much more absorbing energy was lost in the atmosphere than scattering energy (at a very low E of 3.75 hPa).

### 3.5. Albedo Estimations at the TOA and the Surface 

Reflections as well as albedos are key factors affecting radiative transfer, energy balance, and climate [46,47,48,49] and should also be examined to fully understand all of the solar radiation transfer processes and their related energy. The monthly short-wave flux and incoming solar flux at the TOA and the surface under all-sky and clear-sky conditions over a 1° × 1° region (https://ceres.larc.nasa.gov/products.php?product=EBAF-Product, accessed on 1 May 2022) obtained from the Clouds and the Earth’s Radiant Energy System (CERES) Energy Balanced and Filled (EBAF, Edition 4.1) [50,51] were used for albedo calculations and evaluations.

There is a relatively homogeneous surface around the Qomolangma site. An algorithm developed for albedo calculations at the TOA and surface for Sodankylä and QYZ [9,38] was adjusted for Qomolangma. The scattering is assumed to be isotropic at the TOA and the surface; A_0_ expresses the total contribution of the coupled surface–atmosphere at the TOA. Thus, the albedos at the TOA and the surface (Albedo_TOA_ and Albedo_Sur_) were computed using the following equations, respectively:

Albedo_Sur_ = (A_2_e^−AF^ + |A_0_|/e^−AF^)/(A_1_e^−kWm^ × cos(Z) + A_2_e^−AF^) (3)

Albedo_TOA_ = (A_2_ + |A_0_|)/(A_1_ + A_2_) (4)

where e^−AF^ represents the atmospheric transmittance of solar radiation in scattering, and the reflection at the surface was estimated by individual reflection and scattering derived from the TOA (A_0_/e^−AF^). The reflections at the TOA were contributed to by reflection A_0_ and scattering A_2_. The albedo measured by the observations (Albedo_mea_) at the ground was calculated using the ratio of up/downward (G_up_) to upward global solar radiation (G):

Albedo_mea_ = G_up_/G (5)

**Figure 12:**
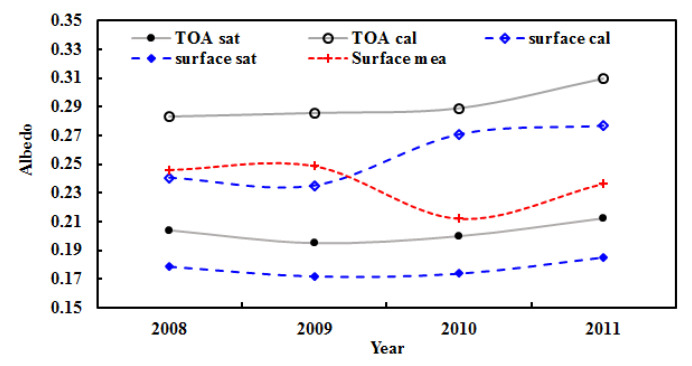
Annual mean albedos during 2008–2011 calculated (cal) and satellite-retrieved (sat) under clear-sky conditions and measured at the surface (mea) at the Qomolangma site.

The estimated mean albedos at the TOA and the surface (n = 7020) during 2008–2011 were 0.293 and 0.234, respectively. The corresponding satellite-derived albedos were 0.203 and 0.177 under clear skies, respectively. Generally, the estimated albedos are in reasonable agreement with the satellite observations, with relative biases of 43.77% and 44.08%. Similarly, the annual mean albedos in each year from 2008 to 2011 were also calculated using hourly measurements, with 0.283, 0.285, 0.289, and 0.310 at the TOA and 0.240, 0.235, 0.271, and 0.277 at the surface (Figure 12). These albedos correspond to the satellite-derived values under clear skies, with relative biases from 38.61% to 46.07% at the TOA and from 34.54% to 55.64% at the surface. The corresponding measured surface albedos were 0.246, 0.249, 0.212, and 0.237 (their average was 0.236) from 2008 to 2011, indicating that the model-estimated surface albedos yielded good estimates with an absolute error of 0.020 and relative bias of 9.16% for the mean surface albedo in 2008–2011. During 2008–2011, the model-estimated and satellite-derived albedos displayed similar variation patterns. For example, the albedos at the TOA and the surface increased by 3.10% and 6.60% for the empirical model estimates, respectively, and 1.48% and 1.22% for the satellite-retrieved values.

**Figure 13:**
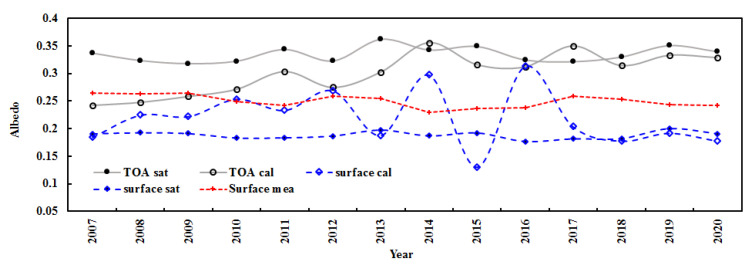
Annual mean albedos during 2007–2020, calculated and satellite-retrieved at the TOA and the surface and measured at the surface (mea) under all-sky conditions at the Qomolangma site.

Similarly, using Equations (3)–(5) and hourly measurements with upward global solar radiation, annual average albedos at the TOA and the surface were calculated for all-sky conditions during 2007–2020 (Figure 13). Generally, the model-estimated and satellite-derived albedos at the TOA and the surface showed small interannual variations. The empirical model underestimated the TOA albedos by 10.10% and overestimated surface albedos by 16.99% compared to the satellite-derived values during 2007–2020. The error of the retrieved albedo is 85.9% using MODIS data in the short-wave region [52].

During 2007–2020, the model-computed and satellite-derived annual albedos increased by 2.85% and 0.27% per year at the TOA, respectively, and decreased by 1.10% and 0.04% per year at the surface, respectively. The measured surface albedos also decreased by 0.50% per year. The annual mean albedos were 0.301 (0.243–0.356) and 0.209 (0.131–0.313) at the TOA and the surface for the model-computed values, 0.335 (0.318–0.362) and 0.188 (0.177–0.200) for satellite-retrieved values, and 0.250 (0.230–0.264) for the surface measured values. In general, the empirical model showed reasonable performance. Both calculated and satellite-derived albedos exhibited similar features, including the finding that albedos at the surface were smaller than those at the TOA.

During 2007–2020, the annual averages for atmospheric GLPs (i.e., AF) decreased by 1.41% per year, the water vapor also decreased by 0.37% per year, the calculated G increased by 0.22% per year, and air temperature decreased by 0.31 °C. Annual losses of G caused by absorbing and scattering GLPs increased by 0.62% and decreased by 2.14%, respectively, and the total loss decreased by 0.08% per year. It seems that the air temperature decline corresponded to a decrease in scattering loss in the atmosphere. It should be noted that to obtain reliable surface albedos, the upward global solar radiation measured in the early morning and late afternoon was not used.

To understand basic atmospheric characteristics at Qomolangma, the annual averages during 2007–2020 were reported: AF = 2.63, E = 4.05 hPa, T = 9.48 °C, RH = 31.11%, v = 3.50 ms^−1^, G_cal_ = 2.36 MJ m^−2^, and G_obs_ = 2.34 MJ m^−2^.

The albedo increases at the TOA in 2007–2020 may be caused by decreases in (1) absorbing and scattering substances or (2) the direct absorption and indirect use of UV and visible radiation by all types of GLPs when reacting with OH radicals and H_2_O [53,54].

The TOA and surface albedos can be computed using radiative transfer models that need more atmospheric parameters (aerosol, cloud, and water properties) [55], and the empirical model in this study is an improvement compared to the current empirical models that cannot output the TOA and the surface albedos (Introduction). More studies on the estimations of the TOA and surface albedos and more accurate ground measurements of global solar radiation are necessary for validation.

## 4. Discussion

### 4.1. Interactions between Changes in Air Temperature and Solar Radiation

Based on reasonable estimations of global solar radiation and albedos at the TOA and the surface, the empirical model can be used to investigate G-GLP interaction. This empirical model is an extended application from the two poles and Qiangyanzhou to Qomolangma, and the detailed mechanisms and meanings of the two terms are fully reported in [9,38,45] (and references therein). In brief, the absorbing term expresses the absorption and utilization of G caused by all absorbing GLPs in the atmosphere through (1) OH, H_2_O, and biogenic volatile organic compounds (BVOCs) in the UV region, (2) excited NO_2_ in the VIS region, and (3) H_2_O, CO_2_, CH_4_, and other absorbers in the NIR region [9,38,53,54,56]. The scattering term expresses multiple scattering by all scattering GLPs (aerosols, clouds, etc.) and multiple reflections between the atmosphere and the land surface.

Air temperature increased at Qomolangma in 2007–2020 (Table 4), associated with increases in G_cal_, G_LA_, and E and a decrease in AF (i.e., a cleaner atmosphere), indicating that all sorts of absorbing gases, liquids, and particles (directly emitted and indirectly produced in the atmosphere, mainly small-sized particles, as AF expresses larger-sized GLPs better than small ones, and AF decreased) should be considered and controlled for reducing regional climate warming in the future. The emissions of all absorbing gases and liquids in the short-wave region (UV, VIS, and NIR) are more important and should be controlled first, comparing the larger increases in E (and G_LA_) to the lower decreases in AF (and G_LS_) (e.g., situation A in Table 4).

### 4.2. Issues about Global Solar Radiation and Its Empirical Model

G_LA_ appeared to be the highest in winter and lowest in spring and summer (Section 3.2), which is mainly because e^−kWm^ × cos(Z) or cos(Z) was larger in summer than in winter, though water-vapor pressure was higher in summer.

The sensitivity analysis (Section 3.4) expresses the responses of global solar radiation to changes in absorbing and scattering substances. The absorbing and scattering losses are caused by absorbing and scattering substances. The annual average absorbing loss was much larger than the scattering loss, which reveals that the absorbing GLP loads in the atmosphere were higher than the scattering GLP loads in the Qomolangma region; i.e., the atmosphere is dominated by absorbing GLPs. Water-vapor pressure was selected as an indicator to represent absorbing GLPs, including NO_2_ and VOCs, as water vapor plays significant roles through different mechanisms in the UV, VIS, and NIR regions. In brief, in the UV region, the OH radical is mainly produced by O_3_ + hv → O_2_ + O(^1^D), O(^1^D) + H_2_O → 2OH. In the visible region, the OH radical is formed by an excited NO_2_* reaction with water molecules. The OH radicals take part in almost all CPRs with GLPs, including NO_x_, SO_2_, VOCs, formic acid, and aerosols, and are recycled. More detailed explanations about the mechanism and roles of OH and water vapor are reported in [9,38,53,54,56] (and references therein). The losses of global solar radiation and the sensitivity test reveal that different features of G resulted from absorbing and scattering GLPs, as well as their changes.

The AF factor expresses the relative absorption and scattering GLP amounts in the atmosphere, and the water-vapor factor expresses the absorbing GLP amounts. When the photochemical and scattering terms are used in the empirical model (Equation (1)), these two terms describe the absorbing and scattering roles, respectively. This can be seen from the weak correlation between the photochemical and scattering terms (R = 0.348, n = 7020, Section 2.2); i.e., global solar radiation can be well distributed into photochemical and scattering terms and processes.

It should be noted that the observational error influences the estimates of global solar radiation; e.g., the values of MAD and RMSE were the smallest for AAVG, increased for MAVG, and then the largest for HAVG with the increase in σ_obs_ (Table 2). Therefore, the estimation errors in annual and monthly mean G evidently decreased with decreases in σ_obs_ (e.g., the RMSE values were 0.58, 0.18, and 0.07 MJ m^−2^ for HAVG, MAVG, and AAVG, respectively, and their corresponding standard deviations σ_obs_ were 1.16, 0.19, and 0.03 MJ m^−2^). Comparing the RMSE (0.31 MJ m^−2^, Table 1), it was close to the mean RMSE (0.22) calculated from the 7 models with the best simulations out of 105 empirical models [34], indicating that the empirical model is acceptable. In more detail, the hourly σ_obs_ values were 0.55, 0.63, and 0.50 MJ m^−2^ in empirical model development for 2008–2011 for Sodankylä, Qomolangma, and Dome C, respectively, and their corresponding RMSE values of hourly G_cal_ were 0.22, 0.31, and 0.04 MJ m^−2^. The largest estimation error was at Qomolangma, and the best observational data at Dome C lay a good foundation for the best estimations of G [12,38,45].

The development and accuracy of the empirical model are based on observed solar radiation and meteorological variables, and good observational data are very necessary. In other words, better performance of G can be achieved when high-quality data are used in model establishment, e.g., for Dome C [45]. Scattering substances, including aerosols (PM_2__.__5_, PM_10_, secondary organic aerosols (SOA), BC, etc.), clouds (in different shapes, amounts, chemical compositions, and ice and/or liquid phases of H_2_O), fog, smog, air pollutants, rain, hail, and especially their changes influence global solar radiation transfer in the atmosphere and at the surface (Section 3.3), indicating that describing all types of scattering GLPs and their scattering processes/roles objectively and accurately is a significant and challenging task for the simulation of G under high GLP loads or cloudy conditions. Therefore, developing a specific empirical model of G for this situation (e.g., S/G ≥ 0.8) is a better option [9]. Comparing the absorbing and scattering GLPs, the absorbing GLPs play more important roles in the three poles and mid-latitude regions. Thus, it is speculated that our atmosphere is dominated by absorbing substances. The empirical model of G is suitable for the Qomolangma region; more studies are needed if this model is applied to other surrounding regions, and the coefficients in the empirical model are required to be validated or adjusted. 

### 4.3. Relationship between Wind Speed and Atmospheric Substances (AF)

**Figure 14:**
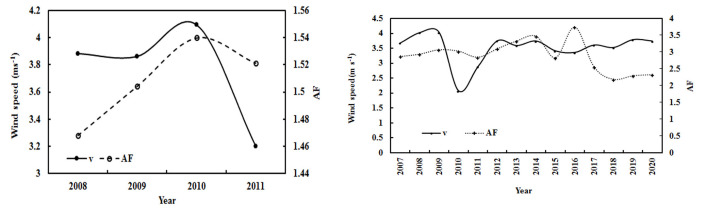
Annual wind speed (v) and atmospheric substances (AF) during 2008–2011 (**left**) and during 2007–2020 (**right**) at the Qomolangma site.

The relationships between wind speed and GLP loads were investigated using hourly data in 2008–2011 (model development, n = 7020). During 2008–2011, annual wind speed decreased by 4.32% per year, and GLPs increased by 1.34% per year (Figure 14). Under all-sky conditions in 2007–2020, annual wind speed increased by 0.39% per year, and GLPs decreased by 1.46% per year. The wind speed and atmospheric GLPs varied inversely under different sky conditions and in different time periods. Negative correlations were found between annual mean v and AF and were 0.06 and 0.10 for the time periods of 2008–2011 and 2007–2020, respectively. A slightly larger negative correlation of 0.17 also existed between monthly mean v and AF in 2007–2020.

Inverse variations and negative correlations between wind speed and atmospheric GLPs (S/G) were also observed at Sodankylä and Qianyanzhou in the Northern Hemisphere [9,38]. However, a positive correlation between v and S/G was found at Dome C [45].

### 4.4. Global Solar Radiation and Other Parameters at Three Polar Sites and a Mid-Latitude Site in 2013–2016

To fully understand global solar radiation and its interactions with its affecting factors, we analyzed global solar radiation and its loss, as well as other related factors at three polar sites and a mid-latitude site, QYZ, China. The areas around the Sodankylä and QYZ sites are mainly covered by boreal coniferous and *Pinus* forests, respectively. The annual averages of monthly G and other parameters were calculated for the four sites under all-sky conditions for 2013–2016 (Table 8). The ratios of all parameters between Sodankylä and QYZ (Ratio 1), Qomolangma and QYZ (Ratio 3), and Dome C and QYZ (Ratio 3) are also given in Table 8.

The estimated G_cal_ × (1-albedo at the surface) (referred to as G’) was 47.75% lower at Qomolangma than QYZ, and the albedo at the TOA was 10.34% larger at Qomolangma than QYZ, causing air temperature to be −15.23 °C lower at Qomolangma than at QYZ. The global solar radiation received at the ground, which can be converted to long-wave radiation that heats the atmosphere (G’), decreased from QYZ to Qomolangma, Sodankylä, and Dome C, closely corresponding to the air temperature decline (Table 8). A higher correlation was found between G’ and T (R = 0.863), indicating that the absorbed radiation at the ground G’ (potential heating energy) plays a significant role in air temperature (or internal energy of the atmosphere, INEA).

The annual averages were analyzed for Sodankylä, Qianyanzhou, and Dome C sites with observed S/G, and positive correlations were 0.903 between mean T and G’ and 0.963 between T and G’ +G_LA_, revealing that scattering energy also makes a small contribution to the increase in air temperature (and INEA).

**Figure 15:**
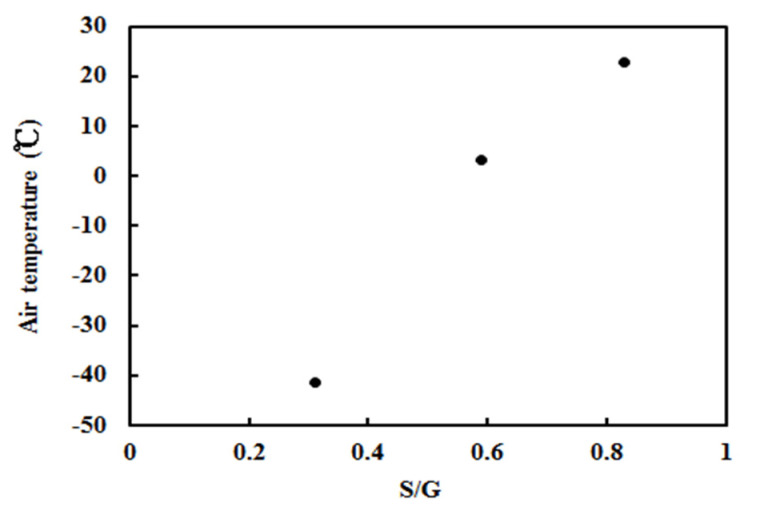
Scatter plot of the annual averages of air temperature and S/G under all-sky conditions at three sites (Sodankyla, Dome C, and Qianyanzhou).

A strong positive correlation existed between mean T and S/G during 2013–2016 for Sodankylä, Qianyanzhou, and Dome C (Figure 15), T = 65.6 × ln(S/G) + 36.012 (R = 0.999), revealing a balanced interaction between net energy in the atmosphere and total atmospheric GLPs during the 4 years. It also reflected the relationship of the equilibrium state of net radiation (short- and long-wave) in the atmosphere–GLP–land system. Using the above equation of T-S/G, the estimated regional maximum and minimum annual air temperatures globally were 36.01 °C and −89.23 °C, corresponding to an S/G of 1.0 to 0.148. The lowest observed annual air temperature was −44.07 °C at Dome C in 2008 (S/G = 0.295).

The annual contributions to energy losses caused by absorbing and scattering GLPs decreased and increased from Dome C to Sodankylä, respectively, implying that the absorbing substances play the most important roles in attenuating solar radiation at the South Pole, followed by the mid-latitude region and then the North Pole. Absorbing GLPs attenuate global radiation more than scattering GLPs at the four sites.

The TOA albedo in the Northern Hemisphere was higher at Sodankylä and then decreased to the lowest at the mid-latitude site. The largest TOA albedo appeared at Dome C (about 0.69). The surface albedos were similar at the three sites in the Northern Hemisphere (0.22), and the largest surface albedo also appeared at Dome C (0.80) because of its snow surface.

During 2013–2016, the annual mean aerosol optical depth (AOD) obtained from MERRA2 aerosol products was the highest at Qianyanzhou, followed by Sodankylä and Qomolangma, and the lowest was at Dome C, which is in good agreement with GLP loads (S/G) (Table 8). The grid value of MERRA2 aerosol products corresponding to the longitude and latitude of the four sites was extracted [57]. A similar feature was also found for the annual AOD at the three poles (Arctic, TP, and Antarctic), which were 0.046, 0.098, and 0.024, respectively [58,59].

The responses of G to absorbing and scattering factors (represented by E and S/G or AF) at the four sites are presented in Table 9 [9,38,45]. At the three poles, G_cal_ was the most sensitive to changes in both the absorbing and scattering factors at Sodankylä, followed by Qomolangma and Dome C, which closely corresponded to the highest absorbing and scattering GLPs at Sodankylä and the lowest values at Dome C (Table 8). All detailed information is shown in Table 8 for a better understanding of the atmosphere and its physical and chemical processes, including the average atmospheric balance states (represented by absorbing and scattering GLPs (E and S/G or AF)), average meteorological variables (T, RH, E, and v; v also indicates kinetic energy of the atmosphere), average solar radiation (G, losses of G, etc.), and their multiple interactions (represented by the ratios to some extent). This indicates that changes in G strongly depend on absorbing and scattering GLPs, as well as their changes. Changes in absorbing or scattering GLPs will lead to different changes in G in the atmosphere and arriving at the ground, together with the reflection at the TOA, and thus different changes in regional climate and climate change (e.g., T and v). More studies on the multiple interactions between solar radiation, GLPs, and climate are very necessary, especially long-term data analysis, including ground and satellite measurements. Besides the commonly implicated absorbers, e.g., GHGs, a large number of absorbers in the UV, VIS, and NIR are suggested for consideration to slow down global warming, for example, N_2_O, black carbon, organic carbon, O_3_, NO_2_, and VOCs ([53,54,55,56,60,61,62,63,64,65,66,67,68] and references therein). Although these gases are transparent and in very low concentrations, their total absorption and indirect use of solar radiation redistribute solar radiation in the atmosphere, at the TOA, and at the surface and change its horizontal and vertical distributions. The relationship between T and S/G at the three poles implies that with more substances (in gas, liquid, and particle phases) in the atmosphere, more energy in the short-wave and long-wave regions is contained and accumulated in the atmosphere, along with higher air temperature. Therefore, the most important task is to restore a cleaner atmosphere to reduce climate warming.

High R_LS_ occurred in spring and summer, especially in summer (Section 3.2), indicating a strong contribution to G by the scattering of small-size aerosols, which are produced by CPRs of GLPs. The emissions of BVOCs are the highest in summer, then decrease in spring and autumn, and are the lowest in winter; combined with high light, high air temperature, and water vapor in summer, lots of SOAs are produced in the Qomolangma region ([65,66,67,68] and references therein). Biomass burning and fluorescence have evident influences on BVOC emissions and O_3_ production [65]. They are the main reasons for high scattering (R_LS_) in summer and spring. Thus, it is recommended to wear long-sleeved shirts and trousers, as well as wide-brimmed hats, for better protection from the high scattering and surface reflection of solar radiation [12].

### 4.5. Solar Radiation and Different Types of Aerosols

**Figure 16:**
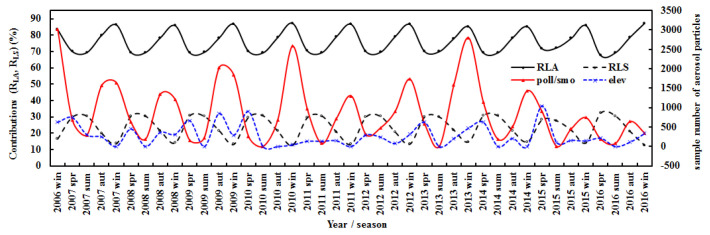
Contributions (R_LA_ and R_LS_) of seasonal absorbing and scattering losses to the seasonal total loss and seasonal averages of sample number of polluted continental/smoke and elevated smoke aerosols (poll/smo, elev) at the Qomolangma site.

To investigate the attenuation effects of different types of aerosols on solar radiation, the contributions of seasonal averages of absorbing and scattering losses (R_LA_ and R_LS_) to the seasonal total loss and the seasonal averages of the number of polluted continental/smoke and elevated smoke aerosols at Qomolangma during 2006–2016 were analyzed (Figure 16).

Based on the nearest interpolation algorithm, the number of samples for each aerosol type in the daytime were extracted from the CALIPSO (Cloud-Aerosol Lidar and Infrared Pathfinder Satellite Observations) Lidar level 3 tropospheric cloud-free aerosol profile product, with a horizontal resolution of 2° (latitude) × 5° (longitude) [69]. During 2007–2016, the sample number of polluted continental/smoke, as well as clean continental aerosols and R_LA_, varied similarly, and the correlations between these two sample numbers and R_LA_ were 0.699 and 0.400, respectively, suggesting that these two types of aerosols were mainly manifested as absorbing features. In addition, elevated smoke aerosols varied similarly with R_LS_, both higher in spring in most situations, but varied similarly with R_LA_ in 2013–2016. The seasonal average in 2007–2016 showed that the total sample number of seven types of aerosols displayed an absorption feature (R = 0.335 between their total number and R_LA_); the combined polluted continental/smoke and polluted dust (82.64% in the seven types of aerosols) resulted in enhanced absorption (R = 0.434). These results reveal that biomass burning is an important aerosol source in spring, which is confirmed by a previous study that reported that biomass burning plumes frequently occurred, especially during spring (March–April) [70]. Different types of aerosols exhibited different absorption and scattering characteristics, which obviously depend on the season (e.g., spring) and sources. More studies on aerosol compositions and optical parameters, as well as their interactions with solar radiation, are needed. Interestingly, according to the MERRA-2 reanalysis data (2000–2021), there was a significant increasing trend of BC at QOMS (Figure S1, Supplementary Material). Absorbing GLPs played dominant roles, and increases in BC and water vapor contributed to the increase in G_LA_ in the Qomolangma region.

## 5. Conclusions

Solar radiation and its transformation processes (absorption, scattering, reflection, etc.), different types of atmospheric substances, and their long-term variations were studied at Qomolangma, the Tibetan Plateau. An empirical model of global solar radiation was developed for Qomolangma, and reasonable estimates under all-sky conditions were calculated. Global solar radiation at the ground and its loss in the atmosphere during 2007–2020 were computed. Sensitivity results revealed that the responses of global solar radiation to changes in absorbing and scattering factors were nonlinear, and global solar radiation was more sensitive to changes in scattering than in absorption. The model-calculated TOA and surface albedos agree with those derived from satellite data. The model-estimated surface albedos are in better agreement with the measured values.

During 2007–2020, the estimated annual global solar radiation decreased by 0.22% per year, associated with a decrease in AF of 1.46% and an increase in E of 0.37% per year. Annual air temperature increased by 0.16 °C. Annual mean absorbing, scattering, and total losses of global solar radiation in the atmosphere were 2.55, 0.64, and 3.19 MJ m^−2^, respectively. The annual absorbing loss increased by 0.42% per year; scattering and total losses decreased by 2.00% and 0.14% per year, respectively. The contributions of annual mean absorbing and scattering losses to the total loss were 77.23% and 22.77%, respectively, indicating that absorbing GLPs play dominant roles. The estimated TOA albedos were larger than surface values. The model-estimated and satellite-derived annual albedos increased at the TOA and decreased at the surface.

Global solar radiation and its absorption, scattering and reflection energy, air temperature, and other critical parameters at the three poles and a mid-latitude site were compared. Global solar radiation and its interactions with GLPs played significant but different roles as driving energy in regional climate and climate change at the three poles. Absorbing GLPs should be controlled to reduce regional climate warming.

## Figures and Tables

**Table 1:** The coefficients and constants (MJ m^−2^), coefficient of determination (R^2^), average and maximum of the absolute relative bias (δavg, δmax (%)), NMSE (MJ m^−2^), and standard deviations of calculated and observed global solar exposure (σ_cal_ and σ_obs_, respectively, MJ m^−2^), along with the mean bias errors (MAD, MJ m^−2^ and %) and the root mean square error (RMSE, MJ m^−2^ and %) (n = 7020).

A_1_	A_2_	A_0_	R^2^	δavg	δmax	NMSE	σ_cal_	σ_obs_	MAD		RMSE	
									(MJ m^−2^)	(%)	(MJ m^−2^)	(%)
5.521	0.859	−1.011	0.712	9.80	56.03	0.01	0.53	0.63	0.26	8.82	0.31	11.38

**Table 2:** Average and maximum of the absolute relative bias (δavg, δmax (%)), NMSE (MJ m^−2^), and standard deviations of calculated and observed global solar exposure (σ_cal_ and σ_obs_, respectively, MJ m^−2^), along with the mean bias errors (MAD, MJ m^−2^ and %) and the root mean square errors (RMSE, MJ m^−2^ and %). HAVG, MAVG, and AAVG are hourly, monthly, and annual averages (G_obs_ ≥ 20 W m^−2^, n = 15,533; G_obs_ ≥ 50 W m^−2^, n = 14,886).

**G_obs_ ≥ 20 W m^−2^**	**δavg**	**δmax**	**NMSE**	**σ_cal_**	**σ_obs_**	**MAD**		**RMSE**	
						**(MJ m^−2^)**	**(%)**	**(MJ m^−2^)**	**(%)**
HAVG	60.14	4525	0.080	1.28	1.16	0.40	19.82	0.58	28.88
MAVG	7.13	24.95	0.008	0.17	0.19	0.14	7.10	0.18	9.10
AAVG	2.72	3.81	0.001	0.05	0.03	0.05	2.72	0.07	3.50
**G_obs_ ≥ 50 W m^−2^**	**δavg**	**δmax**	**NMSE**	**σ_cal_**	**σ_obs_**	**MAD**		**RMSE**	
						**(MJ m^−2^)**	**(%)**	**(MJ m^−2^)**	**(%)**
HAVG	40.07	1576	0.072	1.21	1.13	0.39	18.57	0.56	27.10
MAVG	6.98	25.44	0.008	0.20	0.22	0.14	6.94	0.19	9.00
AAVG	2.63	4.16	0.001	0.03	0.05	0.06	2.62	0.07	3.42

**Table 3:** Same as Table 2, but for the statistical metrics during 2007–2020, using empirical model of global solar radiation (n = 46,683).

Situation	δavg	δmax	NMSE	σ_cal_	σ_obs_	MAD		RMSE	
						(MJ m^−2^)	(%)	(MJ m^−2^)	(%)
HAVG	21.98	357.91	0.048	1.04	0.99	0.35	16.42	0.48	22.13
MAVG	5.46	19.77	0.005	0.23	0.20	0.12	5.49	0.15	7.05
AAVG	2.65	6.42	0.001	0.04	0.06	0.06	2.63	0.07	3.46

**Table 4:** Change rate (%) of the monthly mean solar radiation and its losses and meteorological parameters (air temperature T and its change ΔT (°C), relative humidity (RH), and water-vapor pressure E (hPa) in January 2008–December 2011 (model development, situation A), January 2008–December 2011 (whole measured dataset, situation B), and January 2007 to December 2020 (situation C). The change rate of each variable was calculated using c_1_ × 100/c_0_, and the linear relation between each variable (y) and time (month, x) was determined as y = c_1_x + c_0_.

Situation	G_obs_	G_cal_	T	ΔT (°C)	RH	E	AF	G_LA_	G_LS_	G_L_	n
A	−0.09	−0.05	−0.30	−1.45	0.19	0.86	0.01	0.12	−0.13	0.06	7020
B	−0.16	0.08	−0.19	−0.70	1.06	0.72	0.02	0.07	−0.31	−0.01	14,886
C	−0.001	0.001	0.002	0.47	0	0.008	−0.001	0.003	−0.002	0	46,683

**Table 5:** Correlations among monthly solar radiation (observed and calculated, G_obs_ and G_cal_, losses caused by absorbing and scattering GLPs and total loss, G_LA_, G_LS_, and G_L_) and meteorological parameters (air temperature T, relative humidity RH, water-vapor pressure E, air pressure P, and wind speed v, S/G, in January 2008–December 2011 (model development, n = 7020).

n	T-G_obs_	T-G_cal_	T-G_LA_	T-G_LS_	T-G_L_	T-E	T-AF	G_LA_-E	G_LS_-E	G_L_-E	G_LA_-AF	G_LS_-AF	G_L_-AF	P-AF	v-AF	P-E
7020	0.286	0.284	−0.443	0.455	−0.284	0.569	0.426	−0.130	0.584	0.103	−0.180	0.880	0.173	0.224	−0.145	0.358

**Table 6:** Hourly averages of solar radiation (MJ m^−2^) and meteorological parameters during January 2008–December 2011 (model development, situation A), January 2008–December 2011 (whole measured dataset, situation B), and January 2007 to December 2020 (situation C).

Situation	n	G_obs_	G_cal_	T °C	RH %	E hPa	AF	G_LA_	G_LS_	G_L_	R_LA_ %	R_LS_ %
A	7020	2.99	2.99	9.94	27.50	3.75	1.66	1.76	0.62	2.38	72.54	27.46
B	14,886	2.08	2.11	7.61	30.31	3.55	2.26	2.61	0.65	3.25	77.23	22.77
C	46,683	2.17	2.19	8.03	32.69	3.85	2.97	2.53	0.65	3.18	76.66	23.34

**Table 7:** Change rate of G (%) due to changes in E or AF (%), with AF or E retaining their original values. The change rate of G was computed using (G_caln_ − G_cal_) × 100/G_cal_, where G_caln_ is G_cal_ using new E or AF, and G_cal_ is the previous estimation using the original values of E and AF.

E (%)							AF (%)							
+20	+40	+80	+160	−20	−40	−80	+20	+40	+80	+160	−20	−40	−80	−100
−1.21	−2.28	−4.14	−7.13	1.39	3.05	8.20	−1.35	−2.35	−3.70	−5.09	1.85	4.45	13.74	22.20

**Table 8:** Annual averages of observed monthly meteorological variables and S/G and simulated monthly global solar irradiance and its loss, albedos at the TOA and the surface, AOD, and sample number (n) at Qomolangma (referred to as Qomo), Sodankylä (Sod), Dome C (Dome), and QYZ sites under all-sky conditions during 2013–2016, and the ratios of all parameters between Sodankylä and QYZ, Qomolangma and QYZ, and Dome C and QYZ (ratio 1, ratio 2, and ratio 3) (alb and sur denote albedo and surface, respectively). AF is for Qomolangma only.

Site	G_cal_ MJ m^−2^	T °C	RH %	E hPa	S/G AF	v ms^−1^	G_LA_ MJ m^−2^	G_LS_ MJ m^−2^	G_L_ MJ m^−2^	G’ MJ m^−2^	n	AOD	R_LA_ %	R_LS_ %	alb TOA	alb sur
Sod	0.65	3.05	76.00	6.83	0.59	2.38	1.94	1.23	3.18	0.51	14,343	0.10	61.96	38.04	0.36	0.22
Qomo	2.17	7.48	31.68	3.59	3.35	3.53	2.52	0.68	3.20	0.58	13,424	0.04	76.02	23.98	0.32	0.23
QYZ	1.42	22.71	75.76	22.38	0.83	1.19	1.68	0.27	1.95	1.11	14,915	0.57	89.31	13.69	0.29	0.22
Dome	1.25	−41.39	58.23	0.13	0.31	7.15	3.83	0.18	4.01	0.25	12,412	0.01	95.51	4.49	0.69	0.80
Ratio1	0.46	0.13	1.00	0.30	0.71	2.00	1.15	4.56	1.63	0.46		0.17	0.69	2.77	1.24	0.99
Ratio2	1.53	0.33	0.42	0.16		2.97	1.50	2.52	1.64	0.52		0.08	0.85	1.75	1.10	1.05
Ratio3	0.88	−1.82	0.37	0.006	0.37	6.01	2.28	0.67	2.06	0.23		0.03	1.07	0.33	2.38	3.64

**Table 9:** Change rate of G (%) due to changes in E or S/G (%), with S/G or E retaining their original values. The change rate of G was computed using (G_caln_ − G_cal_) × 100/G_cal_, where G_caln_ is G_cal_ using new E or S/G, and G_cal_ is the previous estimation using original values of E and S/G.

Site	E (%)							S/G (%)							
	+20	+40	+80	+160	−20	−40	−80	+20	+40	+80	+160	−20	−40	−80	−100
Sod	−1.25	−2.37	−4.30	−7.41	1.44	3.17	8.52	−10.03	−18.82	−33.37	−53.69	11.45	24.57	56.93	76.86
QYZ	−2.48	−4.70	−8.53	−14.69	2.86	6.27	16.88	−4.41	−8.22	−14.39	−22.50	5.09	10.96	25.59	34.65
Dome	−0.48	−0.91	−1.66	−1.85	0.56	1.22	3.28	−0.89	−1.76	−3.42	−6.48	0.92	1.86	3.84	4.87

## Data Availability

Not applicable; solar radiation and meteorological data at QOMS Station were obtained from https://data.tpdc.ac.cn/zh-hans/data/b9ab35b2-81fb-4330-925f-4d9860ac47c3/ (accessed on 1 January 2022).

## Supplementary Materials

The following supporting information can be downloaded at: https://www.mdpi.com/article/10.3390/ijerph19158906/s1, Figure S1: Black carbon concentration in 2000–2021 at QOMS station from monthly MERRA-2 data.
