# Supplementary material for: Long-Term Variations in Global Solar Radiation and Its Interaction with Atmospheric Substances at Qomolangma
Source: Int J Environ Res Public Health. 2022 Jul 22;19(15):8906. doi: 10.3390/ijerph19158906 (PMC9332281; doi:10.3390/ijerph19158906)
Supplement: Supplementary file 1 [file ijerph-19-08906-s001.zip › ijerph-1775441-supplementary.pdf]

supplementary: list of acronyms and Figure S1

list of acronyms

AAVG	annual averages
AF	attenuation factor
AOD	aerosol optical depth
BC	black carbon
BVOCs	biogenic volatile organic compounds
CERES	Clouds and the Earth's Radiant Energy System
CPRs	chemical and photochemical reactions
GHGs	greenhouse gases
GLPs	gases, liquids, and particles
HAVG	hourly average
INEA	internal energy of the atmosphere
IPCC	Intergovernmental Panel on Climate Change
MAVG	monthly average
NIR	near infrared radiation
SOA	secondary organic aerosols
TOA	top of the atmosphere
UV	ultraviolet radiation
VIS	visible radiation
VOCs	volatile organic compounds

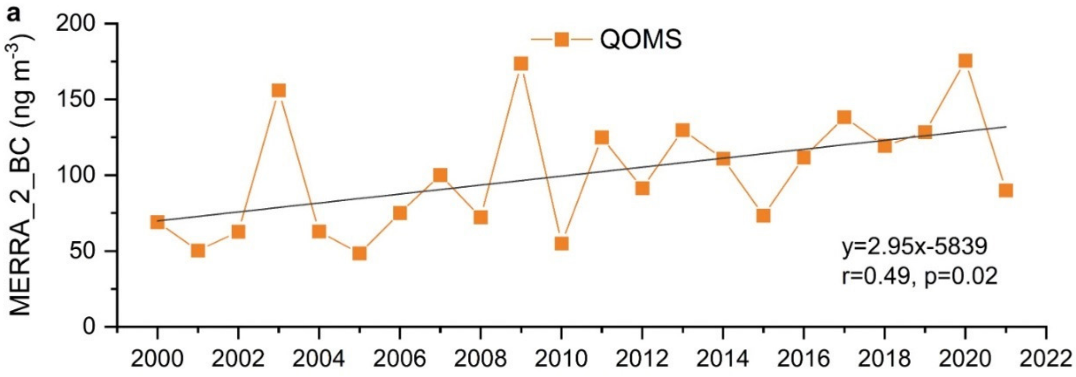

Figure S1. Black carbon concentration in 2000-2021 at QOMS station from monthly MERRA-2 data.
